# Neither the HIV Protease Inhibitor Lopinavir-Ritonavir nor the Antimicrobial Trimethoprim-Sulfamethoxazole Prevent Malaria Relapse in *Plasmodium cynomolgi*-Infected Non-Human Primates

**DOI:** 10.1371/journal.pone.0115506

**Published:** 2014-12-26

**Authors:** Charlotte V. Hobbs, Saurabh Dixit, Scott R. Penzak, Tejram Sahu, Sachy Orr-Gonzalez, Lynn Lambert, Katie Zeleski, Jingyang Chen, Jillian Neal, William Borkowsky, Yimin Wu, Patrick E. Duffy

**Affiliations:** 1 Laboratory of Malaria Immunology and Vaccinology, National Institute of Allergy and Infectious Diseases, National Institutes of Health, Rockville, Maryland, United States of America; 2 Department of Pharmacotherapy, University of North Texas System College of Pharmacy, Fort Worth, Texas, 76107, United States of America; 3 Department of Pediatrics, Division of Infectious Disease and Immunology, New York University School of Medicine, New York, New York, United States of America; University of British Columbia, Canada

## Abstract

*Plasmodium vivax* malaria causes significant morbidity and mortality worldwide, and only one drug is in clinical use that can kill the hypnozoites that cause *P. vivax* relapses. HIV and *P. vivax* malaria geographically overlap in many areas of the world, including South America and Asia. Despite the increasing body of knowledge regarding HIV protease inhibitors (HIV PIs) on *P. falciparum* malaria, there are no data regarding the effects of these treatments on *P. vivax*'s hypnozoite form and clinical relapses of malaria. We have previously shown that the HIV protease inhibitor lopinavir-ritonavir (LPV-RTV) and the antibiotic trimethoprim sulfamethoxazole (TMP-SMX) inhibit *Plasmodium* actively dividing liver stages in rodent malarias and in vitro in *P. falciparum*, but effect against *Plasmodium* dormant hypnozoite forms remains untested. Separately, although other antifolates have been tested against hypnozoites, the antibiotic trimethoprim sulfamethoxazole, commonly used in HIV infection and exposure management, has not been evaluated for hypnozoite-killing activity. Since *Plasmodium cynomolgi* is an established animal model for the study of liver stages of malaria as a surrogate for *P. vivax* infection, we investigated the antimalarial activity of these drugs on *Plasmodium cynomolgi* relapsing malaria in rhesus macaques. Herein, we demonstrate that neither TMP-SMX nor LPV-RTV kills hypnozoite parasite liver stage forms at the doses tested. Because HIV and malaria geographically overlap, and more patients are being managed for HIV infection and exposure, understanding HIV drug impact on malaria infection is important.

## Introduction

*Plasmodium vivax* malaria causes significant morbidity and mortality worldwide [1]. HIV and *Plasmodium vivax* malaria geographically overlap in many areas of the world, including South America and Asia, and impact of drugs used in HIV management on *P. vivax* remains understudied. In addition, treatment of *P. vivax* is complicated by the ability of the parasite to assume a hypnozoite form in the liver stages, which leads to relapsing malaria [2]–[4].

The World Health Organization (WHO) recommends HIV management with combination antiretroviral therapy (ARV), with first line therapy including a non-nucleoside reverse transcriptase inhibitor (NNRTI) and 2 nucleoside reverse transcriptase inhibitors (NRTIs) with few exceptions, and second line therapy including an HIV protease inhibitor (HIV PI) and 2 NRTIs [5]. We have previously shown that HIV PIs kill actively dividing liver stage forms in rodent malaria models and in *P. falciparum in vitro*, whereas NNRTIs do not [6], [7]. In addition, HIV PIs have been shown to kill *P. vivax* in *ex vivo* culture [4]. We and others have demonstrated that HIV PIs kill malaria parasites in asexual and sexual blood stages, and they can block transmission, in vitro [8]–[16]. In addition, a recent clinical study showed a reduction in recurrent malaria events with the use of the HIV PI lopinavir-ritonavir (LPV-RTV) in an area of high transmission intensity where *P. falciparum* malaria is prevalent [17], with a reduced risk of gametocytemia in HIV PI-treated patients on the day of malaria diagnosis as well as 28 days later [18]. Separately, many clinical studies have shown the antibiotic, trimethoprim-sulfamethoxazole (TMP-SMX), used to prevent opportunistic infections in HIV-exposed infants and HIV-infected patients [5] can reduce clinical malaria burden [3], and we have previously shown that TMP-SMX blocks development of dividing liver stage *Plasmodium* parasites in rodent malaria models [19] and in *P. falciparum in vitro* [19]. However, whether LPV-RTV or TMP-SMX kill liver stage hypnozoites, or dormant liver stage form responsible for malaria relapse, has not been tested. Drug effect on the hypnozoite is important to investigate as this form perpetuates the cycle of transmission. Indeed, the only clinically available drug which can target this stage is primaquine, which cannot be used in patients with glucose 6 phosphate dehydrogenase (G6PD) deficiency or in pregnant women, and which requires a 14 day course to kill hypnozoite forms in the liver [20].

Given the overlap of HIV and malaria with HIV-infected and exposed patients in endemic areas receiving these drugs, and given the shortage of drugs available that offer radical cure for *P. vivax* malaria, we set out to investigate the effects of the HIV PI lopinavir (here, LPV-RTV), the most potent antimalarial HIV PI with other *Plasmodium* species [6], [12], and TMP-SMX, on *P. cynomolgi* liver stage hypnozoite forms, an established animal model for *P. vivax* [20].

## Materials and Methods

### Non-Human Primates

All studies described herein were performed on an animal study proposal approved by the NIAID Institutional Animal Care and Use Committee. Macaca mulatta (rhesus macaque) monkeys of Indian origin maintained in facilities accredited by the Association for Assessment and Accreditation of Laboratory Animal Care were used, and all experiments were performed in accordance with the guidelines of the NIAID Division of Intramural Research (DIR) Animal Care and Use Committee (Permit Number ASP LMIV 9E). For the pilot experiments (summarized in Table 1), 3 male monkeys were used with weight ranging from 8.1–9.36 kg. Six male and 3 female animals were used for the experiment testing drug effects, with weight ranging from 4.94 to 10.34 kg. Animals were observed at least twice per day with abnormalities reported to the staff veterinarian by the end of the observation period. Animals were gavaged and infected under sedation with ketamine (0.1 cc/kg 10% Ketamine intramuscularly, or IM), with close monitoring for clinical and behavioral changes, and all efforts were made to minimize suffering. Our study endpoint was the detection of parasites on malaria smear, at which time animals were immediately treated (<1% parasitemia).

**Table 1:** Treatment with Quinidine gluconate in Rhesus Monkeys: Dose: 26 mg/kg IM Twice Per Day x 7 days.

Infection with *P.cynomolgi*	Result
Frozen vial infected red blood cells* (Animal CL4K)**	Parasite detected D4, treated, smear negative for 6 weeks
Frozen vial infected red blood cells at 0.9% parasitemia x2 on D0 and D7 (Animal CL4K, donor)**	Parasite detected D9, treated, smear negative for 6 weeks
Infected-Mosquito bites (Animal DBOB)	Primary attack D9: treated with quinidine; relapse #1 D29, #2 D46
Infected-Mosquito bites (Animal DB9P)	Primary attack D11: treated with quinidine; relapse #1 D29, #2 D45

*Kind gift of Bill Collins, PhD to Bob Gwadz, PhD.

**these experiments demonstrate curative efficacy of regimen in curing asexual blood stages since there is no possibility of relapse.

### Monitoring and Treatment of Malaria Infections (Fig. 1)

This relapse study model was conducted as previously with few modifications [20]–[24]. No monkeys used in this study were previously involved in other malaria vaccination or antimalarial drug testing studies, and monkeys had baseline CBC and chemistries (including liver function tests) that were within normal limits two weeks preceding the study. All drugs used were commercially available and purchased, except quinine dihydrochloride (kind gift of Dr. Jetsumon Sattabongkot Prachumsri, Mahidol University, Bangkok, Thailand).

Quinine dihydrochloride dosing was based on a mg/kg regimen used at the United States Army Materiel Command-United States Army Armed Forces Research Institute of Medical Sciences (USSAMC-AFRIMS, Bangkok, Thailand) (Dr. Imerbsin Rawiwan, United States Army Material Command (pers. comm.)), where quinine 20 mg/kg base dose IM BID for 7 days with a single dose of artesunate 8 mg/kg IV times one is used for high parasitemias. From this, we derived a dose of 32 mg/kg quinine dihydrochloride (conversion based on approximation of clinical converting recommendations [25]), twice daily by intramuscular (IM) injection for 5 days [26] to clear asexual parasite forms, and to avoid possible pharmacokinetic interactions between quinine and LPV-RTV [27]–[30], Quinidine gluconate dosing was based on the quinine base 20 mg/kg regimen and 26 mg/kg quinidine gluconate IM twice per day for 7 days was used (conversion based also on approximation of clinical converting recommendations [31]).

Prior to the below experiment, pilot studies using *P. cynomolgi* infected red blood cell stock were originally provided by Dr. Bill Collins (Centers for Disease Control and Prevention, Atlanta, Georgia, USA), or from our own prepared stocks, were used to infect a splenectomized monkey to test the quinidine gluconate regimen used as described below. This monkey also served as a donor (as described below) for a mosquito bite infection study in which the quinidine regimen used was also tested. In the former, the splenectomized monkey was infected and tested positive for parasites by D4–D9 after infection and was cured with quinidine gluconate 26 mg/kg IM BID x 7 days with smears remaining negative for 6 weeks. In the latter, two monkeys were infected by the bites of mosquitoes and one had a primary attack D9, relapse #1 D29, #2 D46; the second had Primary attack D11, relapse #1 D29, #2 D45 (Table 1).

To produce infected mosquitoes, laboratory-reared *Anopheles stephensi* mosquitoes (Laboratory of Malaria Vector Research, NIAID) were allowed to feed on a splenectomized donor rhesus monkey that had been infected with *P. cynomolgi* B strain by intravenous (IV) injection of 1×10^6^ frozen infected RBCs (derived from frozen vial of 0.9% parasitemia) thawed from frozen stock. When mature sporozoites reached the salivary glands at day 10 to 12, the infected mosquitoes were allowed to feed on 9 malaria-naive monkeys for 30 min, with 34 to 51 bites recorded used to infect each monkey (Day 0, or “D0”  =  Day of infection of test monkeys with sporozoites). After feeding, mosquitoes not engorged with blood were removed, and mosquito bite counts represent the number of mosquitoes with blood (Table 2). Approximately 7 to 8 days after feeding, parasites were detected in peripheral blood, and the monkeys were treated with quinine dihydrochloride (32 mg/kg) twice daily by intramuscular (IM) injection for 5 days to clear all asexual parasite forms, thereafter presumably leaving only hypnozoite forms in the monkeys' livers since quinine (and quinidine) only kill blood stage parasite forms [20], [21].

**Table 2:** Bites Counted Per Monkey in Experiment Assessing Effects of the HIV Protease Inhibitor Lopinavir-ritonavir and the Antimicrobial Trimethoprim-Sulfamethoxazole on Relapse in *Plasmodium cynomolgi*-infected Rhesus Monkeys.

Group	Number of Bites
Control: EZD, DB36, D89H	51, 34, 24
TMP-SMX: DC04, DBZ8, DA8G	40, 33, 16
LPV-RTV: CL84, FLM, FZ8	34, 30, 21

Nine monkeys were divided into three groups after completion of quinine therapy, which was used to cure the primary attack due to concerns for drug interaction with a higher potential frequency of cardiac side effects if quinidine had been used immediately preceding LPV-RTV [27]–[29], [32]. All drugs were administered by gavage; the third group received no additional treatment after quinine therapy and served as a control. Test drugs were administered after quinine to test the effect of drug on hypnozoites, which would remain after quinine treatment. TMP-SMX was administered as 4 mg/kg TMP +20 mg/kg of SMX twice daily orally derived from prophylaxis dosing information for *Pneumocystis jirovecii* [33]. LPV-RTV was administered as12 mg/kg lopinavir +3 mg/kg ritonavir twice daily orally based on a prior study in which this dose was deemed safe in monkeys [34]. Monkeys received TMP-SMX for 4 days starting on D17 or LPV-RTV for 7 days, starting on D21. Parasitemia was monitored daily to observe relapse patterns. Due to shortage of quinine, quinidine gluconate treatment (26 mg/kg twice daily for 7 days IM [21]) was applied to all groups when the first relapse was observed in the control group once the animals reached 1%, and one control animal (DB9H) received a dose of artesunate 8mg/kg IV times one due to a rapid rise in parasitemia (from 0.55% to 4.75%) within two smear readings. At the end of the study, all monkeys received radical cure by gavage with primaquine 0.5 mg base/kg once per day for 14 days and chloroquine 25 mg base/kg once per day for 3 days after the second relapse in the control group [20]–[24].

Giemsa stain thin smears were monitored during the study daily once parasites were observed to every other day if parasitemia had resolved (up to D90, to ensure radical cure), and 10,000 red cells per smear were examined before being declared negative.

Based on the pharmacokinetic profiles of the drugs used in this investigation, and the assumption that 4–5 half-lives are required to both reach steady state and for drug elimination to occur [29], [35]–[37], plasma concentrations were assumed to reach steady state as well as for drug elimination to occur during a period in which hypnozoites would be present [21], [26], [38]. Pharmacokinetics of TMP and SMX parallel what is seen in humans [35]. Plasma concentrations of LPV and RTV were expected to be below clinically relevant concentrations [39].

### Study Endpoints

An increase in the time to detection of parasites in the presumed first and second relapses as detected by smear were each used to deduce liver stage reductions, and appearance of parasites after treatment with quinine and quinidine were considered relapses, as previously described [20], [23]. Study outcomes are presented as Kaplan-Meier survival curves, and statistics are descriptive [40].

## Results

By smear, all control monkeys had time to first detection of blood stage parasites of 9–12 days (first parasitemias detected on D9–12 post infection) (Fig. 2). Only 2/3 control monkeys had detectable blood stage parasites (presumed first relapse) by smear by D22. All 3 monkeys did have another (second) relapse on D39, D39, D44.

The TMP-SMX-treated monkeys did not have a first relapse at all, but did have second relapses Day 43, D41, D45.

LPV-RTV-treated monkeys all had first and second relapses which occurred at parallel time points (1st relapse  = D25, D22, D22; 2nd relapse: D37, D40, D42) compared with the control monkeys, suggesting that there was no impact on hypnozoites at this dose.

## Discussion

We have demonstrated that TMP-SMX and LPV-RTV do not have significant effect on relapse in the *P. cynomolgi* rhesus macaque model. This is the first evaluation of LPV-RTV and TMP-SMX *in vivo* in non-human primates against relapses of *P. cynomolgi* as a model for *P.vivax*.

Time to first detection of blood stage parasites, or prepatent period, was 9-12 days in 2 control monkeys, consistent with previous reports [22], [26]. Although only 2/3 control monkeys had detectable blood stage parasites (presumed first relapse) by smear by D22, all 3 monkeys had relapses on D39, D39, D44. The absence of first relapse (in one animal) and variability of parasitemia is within the relapse response range observed in other reports [26].

LPV-RTV did not increase prepatent period, nor did it prevent relapse. LPV-RTV-treated monkeys all had first and second relapses which occurred at parallel time points (1st relapse  = D25, D22, D22; 2nd relapse: D37, D40, D42) compared with the control monkeys, suggesting that there was no impact of the HIV PI on hypnozoites. HIV PIs have been shown to kill a multitude of *Plasmodium* species and life cycle stages, including *P. vivax* ex vivo [4], as well as actively dividing liver stage rodent malaria parasites [6] and *P. falciparum* [7]. Although the mechanism by which the HIV protease inhibitors kill the malaria parasite remains unknown, it is hypothesized that they interfere with aspartyl protease, or plasmepsin, function in the malaria parasite [7]. It is possible that, although it is not known if these or one of these proteases is the target of HIV PIs in malaria, the target may not be important or active in the hypnozoite stage. Indeed the metabolic roles of the plasmepsins, especially in *P. vivax*, have not been fully described [41], [42]. Of note LPV-RTV dosing as used in this study would achieve levels far below clinical significance [39]. Thus it is also possible that the doses used of the HIV PIs were not high enough to exert antimalarial effect.

The TMP-SMX-treated monkeys did not have a first relapse at all, but did have relapses Day 43, D41, and D45. As for TMP-SMX, delay to the first appearance of parasitemia but no difference between relapse in treated compared with control suggests there was killing of the actively dividing liver stage parasites, but not hypnozoites [43]. Antifolate-killing of actively dividing liver stage parasites is consistent with data we have previously published in rodent malaria models, in *P. falciparum in vitro,* and in *P. knowlesi in vivo* [19], [39]). These data are also consistent with prior work in which it has been demonstrated that other antifolates such as pyrimethamine and proguanil, when administered between the time of sporozoites inoculation and invasion of the blood, may simply eliminate growing liver schizonts and thereby delay primary parasitemia, that is, increase prepatent period, which directly correlates to reduction of actively dividing parasites in the liver [43]. If hypnozoites are spared, however, which they have been shown to be with pyrimethamine and proguanil with *P. cynomolgi in vivo*, primary parasitemia itself could be delayed and relapses still occur. These antifolates have also been shown to kill the exoerythrocytic stages of *P. cynomolgi* and *P. knowlesi in vitro* [40], [44]–[48], all consistent with what we observed. Trimethoprim alone, and at a much higher dose (50 mg/kg BID compared to the 4 mg/kg BID we used) has been shown to have no anti-hypnozoite effect, nor an effect on delaying primary parasitemia, when used in combination with sub-therapeutic doses of primaquine [48]. Reduction of liver stage burden may translate to delay in blood stage infections in the field, but absence of anti-hypnozoite effect in *P. cynomolgi* indicates TMP-SMX would offer no benefit in treating or preventing *P. vivax* malaria.

An alternative explanation is that delay to the first appearance of parasitemia in this TMP-SMX treated group could be due to incomplete effectiveness of a shortened regimen of quinine fully killing asexual stage parasites, with TMP-SMX killing asexual stages. Other antifolates can kill *P. cynomolgi* asexual stage [49].

**Figure 1:**
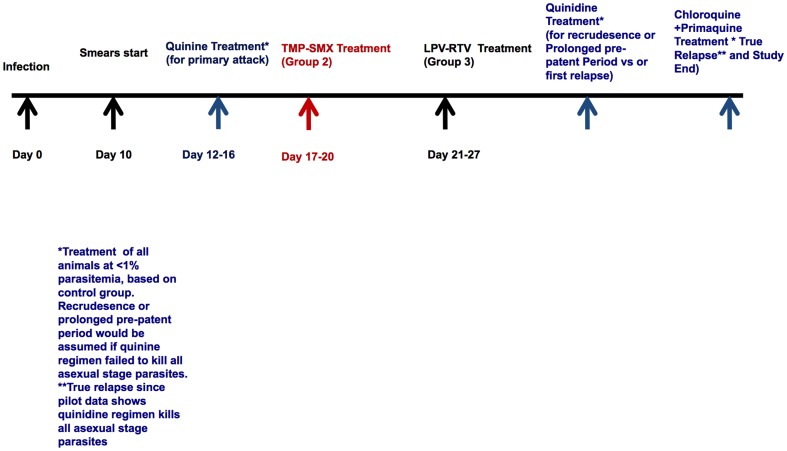
Drug Efficacy Against Relapse Study Schematic. On Day 0, all monkeys were infected by with bites of *P. cynomolgi*-infected mosquitoes. Quinine therapy was administered at parasitemia <1%, to clear all asexual parasite forms, thereafter leaving only hypnozoite forms in the monkeys' livers. After parasitemia returned to zero and once the drugs were presumed safely eliminated, monkeys received trimethoprim-sulfamethoxazole (TMP-SMX), lopinavir-ritonavir (LPV-RTV), or no additional drug administration (control). Parasitemia was then monitored daily for relapse, and quinidine treatment was administered to all groups when the first relapse was observed in the control group. Monkeys received primaquine and chloroquine for radical cure after the second relapse in the control group.

In either case, the parasites seen on D43, 41, 45 are relapses because all animals were treated for the first parasitemia observed after the primary attack with quinidine, and this regimen was proven effective at eradicating asexual stages in our pilot experiments (Table 1). Therefore, based on our pilot data, what we call the “second relapse” we believe is a true relapse since we demonstrated our quinidine was effective at killing asexual blood stages (Fig. 1 and Table 1).

**Figure 2:**
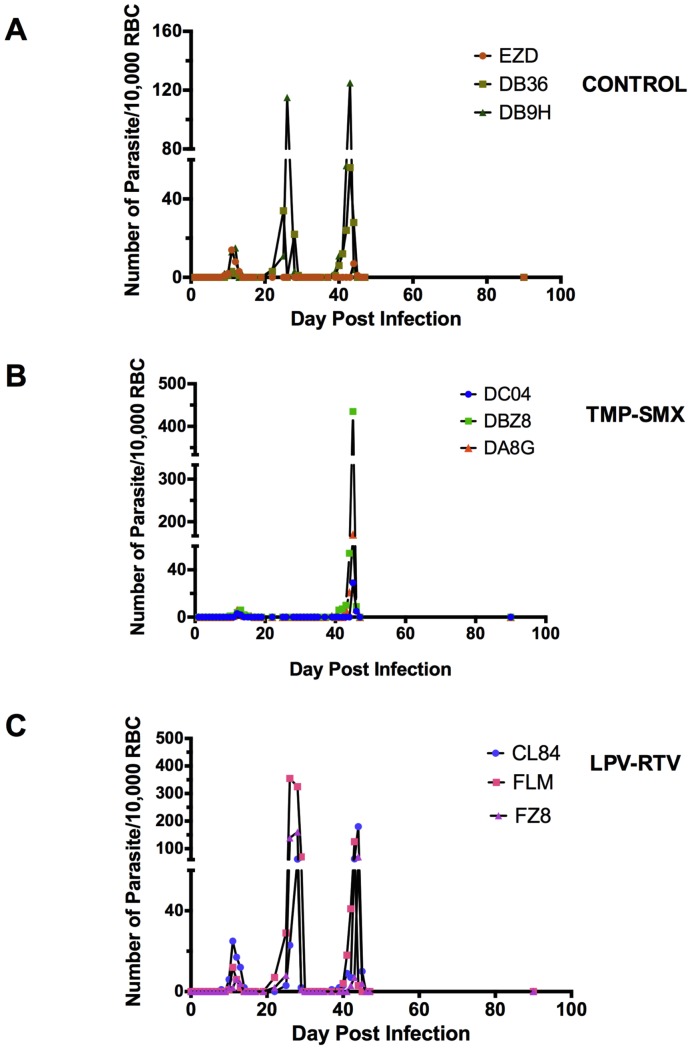
TMP-SMX and LPV-RTV plus TMP-SMX failed to prevent or delay *P.cynomolgi* relapses at given doses. Relapse patterns in *Plasmodium cynomolgi* B strain infected rhesus monkeys after 5 days of quinine treatment to clear the initial parasitemia, as outlined in Fig. 1. Panels are as follows: (A) control monkeys, treated with quinine only; (B) monkeys treated with quinine and then with 4 mg/kg of TMP +20 mg/kg of SMX of commercially available suspension twice per day D17–20 (C) monkeys treated with quinine and then with lopinavir-ritonavir (LPV-RTV) 12 mg lopinavir; 3 mg ritonavir/kg D21–27. Smears were obtained as outlined in Fig. 1.

Because HIV and malaria overlap, further studies are required to validate whether PIs and TMP-SMX offer benefit in reducing clinical malaria through anti-liver stage, although possibly not anti-hypnozoite, effect. If drugs used in HIV management can reduce malaria burden, their administration can be tailored or timed to maximize those benefits.
